# Effects of dietary supplementation with Taiwanese tea byproducts and probiotics on growth performance, lipid metabolism, and the immune response in red feather native chickens

**DOI:** 10.5713/ajas.20.0223

**Published:** 2020-08-21

**Authors:** L. W. Chen, W. Y. Chuang, Y. C. Hsieh, H. H. Lin, W. C. Lin, L. J. Lin, S. C. Chang, T. T. Lee

**Affiliations:** 1 Department of Animal Science, National Chung Hsing University, Taichung, 402, Taiwan; 2 School of Chinese Medicine, College of Chinese Medicine, China Medical University, Taichung, 404, Taiwan; 3 Kaohsiung Animal Propagation Station, Livestock Research Institute, Council of Agriculture, 912, Taiwan; 4 The iEGG and Animal Biotechnology Center, National Chung Hsing University, Taichung, 402, Taiwan

**Keywords:** Green Tea By-product, Red Feather Native Chicken, Anti-obesity, Lipid Metabolism, Anti-inflammation

## Abstract

**Objective:**

This study compared the catechin composition of different tea byproducts and investigated the effects of dietary supplementation with green tea byproducts on the accumulation of abdominal fat, the modulation of lipid metabolism, and the inflammatory response in red feather native chickens.

**Methods:**

Bioactive compounds were detected, and *in vitro* anti-obesity capacity analyzed via 3T3-L1 preadipocytes. In animal experiments, 320 one-day-old red feather native chickens were divided into 4 treatment groups: control, basal diet supplemented with 0.5% Jinxuan byproduct (JBP), basal diet supplemented with 1% JBP, or basal diet supplemented with 5×10^6^ colony-forming unit (CFU)/kg *Bacillus amyloliquefaciens*+5×10^6^ CFU/kg *Saccharomyces cerevisiae* (BA+SC). Growth performance, serum characteristics, carcass characteristics, and the mRNA expression of selected genes were measured.

**Results:**

This study compared several cultivars of tea, but Jinxuan showed the highest levels of the anti-obesity compound epigallocatechin gallate. 3T3-L1 preadipocytes treated with Jinxuan extract significantly reduced lipid accumulation. There were no significant differences in growth performance, serum characteristics, or carcass characteristics among the groups. However, in the 0.5% JBP group, mRNA expression of fatty acid synthase (FAS) and acetyl-CoA carboxylase (ACC) were significantly decreased. In the 1% JBP group, FAS, ACC and peroxisome proliferator-activated receptor γ levels were significantly decreased. Moreover, inflammation-related mRNA expression levels were decreased by the addition of JBP.

**Conclusion:**

JBP contained abundant catechins and related bioactive compounds, which reduced lipid accumulation in 3T3-L1 preadipocytes, however there was no significant reduction in abdominal fat. This may be due to a lack of active anti-obesity compounds or because the major changes in fat metabolism were not in the abdomen. Nonetheless, lipogenesis-related and inflammation-related mRNA expression were reduced in the 1% JBP group. In addition, dietary supplementation with tea byproducts could reduce the massive amount of byproducts created during tea production and modulate lipid metabolism and the inflammatory response in chickens.

## INTRODUCTION

As modern consumers are pursuing a healthy low-fat diet, meat which accumulates excessive fat is not desirable [1]. In the past, scientists have focused on breeding and nutrition to improve the growth rate of animals, but this was accompanied with an accumulation of excessive fat which not only affected the animals’ health but also reduced carcass percentage.

Red feather native chickens are indigenous to Taiwan and have been bred for decades. Although popular due to its tasty meat, its excessive fat content may increase consumers’ health concerns and reduce purchase intention. It is therefore beneficial to reduce fat accumulation in red feather native chickens.

Tea, made from the plant *Camellia sinensis*, includes green tea, oolong, and black tea. Each has a different degree of fermentation but green tea contains the most catechins [2]. More than three million tons of tea are grown worldwide every year, and green tea has long been a popular drink, especially in Asia [3]. Green tea production is massive, and as a result there is a large amount of byproduct (green tea powder) from tea that is broken during production. While the source of green tea powder is stable, it has no practical application except as compost or general waste. However, green tea has several beneficial properties, including anti-obesity, anti-inflammatory, antibacterial, and antioxidative effects [4–6], which may be due to its abundant phenols, including catechins and flavonoids [7]. Furthermore, epigallocatechin gallate (EGCG), the major catechin in green tea and its most bioactive component, has demonstrated an anti-obesity effect on cells in animal studies [8]. In this study, byproducts of Oolong, Yingxiang, Jinxuan, Longjing, Biluochun, and Black tea have been compared the content bioactive compounds, and the tea byproducts in this study are slightly fermentation except black tea is highly fermentation.

Probiotics have been demonstrated benefit on poultry by the properties of antimicrobial and immune function regulation. Dietary supplementation with probiotics in broilers improved growth performance and intestinal morphology [9]. These findings are similar to our previous study on probiotic applications [10], which reported a positive effect on intestinal morphology when broilers were fed *Bacillus amyloliquefaciens* (BA) and *Saccharomyces cerevisiae* (SC). Moreover, probiotics may regulate the pathway of fat metabolism by alter the properties of resident microbiota in the gut [11]. Therefore, BA+SC have been supplemented for red feather native chickens in this study to evaluate the effects on growth performance, lipid metabolism, and the immune response.

Whether *in vitro* or *in vivo*, the anti-obesity effect of green tea and EGCG have been widely studied and applied, but the relationship between obesity and the inflammatory response in broilers and the underlying molecular mechanisms require further research. As such, this study used green tea byproducts and probiotics as feed additives for red feather native chickens, with the expectation that it would reduce the accumulation of abdominal fat and regulate the inflammatory response. The anti-obesity ability of green tea byproducts was first investigated via 3T3-L1 preadipocytes, which have been widely employed to study adipocyte differentiation and evaluate lipid accumulation. We then studied the effects of green tea byproduct and probiotic supplementation on growth performance, percentage of abdominal fat, and serum characteristics in red feather native chickens. Due to the close relationship between obesity and inflammation, the mRNA expressions of lipid metabolism and inflammation were also analyzed.

## MATERIALS AND METHODS

### Preparation of Jinxuan extracts for cell culture, and measurement of total phenols and total flavonoids

Jinxuan byproduct (JBP) was extracted using deionized water at 95°C for 3 h then diluted with deionized water, before filtration through a 0.22 μm membrane filter. The filtrate was stored at −20°C for later analysis.

### High-performance liquid chromatography assay

Gallocatechin (GC), epigallocatechin (EGC), catechin (C), epicatechin (EC), EGCG, gallocatechin gallate (GCG), epicatechin gallate (ECG), gallic acid (GA), and caffeine were identified via high-performance liquid chromatography (HPLC) with a column (ACE Excel 5 C18-AR, 250 mm×4.6 mm), and column oven set at 40°C. The HPLC system consisted of a pump (CM 5110), an autosampler (CM 5210), a column oven (CM 5310), a UV-VIS detector (CM 5420), and analysis software (Chromaster system manager, version 1.0) set to the manufacturer’s protocols (HITACHI, Kyoto, Japan). Samples were extracted and diluted with methanol, then filtered through a 0.22 μm membrane filter. The conditions for the mobile phase were 0.05% v/v H_3_PO_4_ (A) and CH_3_OH/CH_3_CN solution (B) (3:2, v/v) with a flow rate of 1.0 mL/min and the detection wavelength set at 280 nm. The gradient program was: 90% (A) and 10% (B) at 0 min; 75% (A) and 25% (B) at 15 min; 40% (A) and 60% (B) at 25 min; 90% (A) and 10% (B) at 25.1 min; and 90% (A) and 10% (B) at 40 min. Sample peaks were identified by comparing retention times. The results were calculated via standard curve using the reference standards. Final data was expressed as mg/g dry matter (DM).

### Total phenolic content

The total phenolic content was determined using a Folin–Ciocalteu reagent according to the methods of Kujala et al [12]. Briefly, Folin–Ciocalteu reagent was mixed with JBP and 7.5% sodium carbonate then allowed to react for 30 min at room temperature (RT). Absorbance was measured at 730 nm with a microplate reader (Sunrise, Tecan, Männedorf, Switzerland), and the equation from the GA standard curve was used to determine the phenolic compounds of JBP. Total phenolic content was expressed as GA equivalents (mg gallic acid equivalent [GAE]/g DM).

### Total flavonoid content

The flavonoid content of JBP was determined via the colorimetric method. Briefly, JBP was mixed with methanol, 10% aluminum chloride (AlCl_3_), and 1 M potassium acetate then left at RT for 30 min. The absorbance of the reaction mixture was measured at 415 nm with a microplate reader (Sunrise, Tecan, Switzerland), and the calibration curve was obtained by preparing quercetin solutions. The flavonoid content was expressed as micrograms of quercetin equivalent (mg quercetin equivalent [QE]/g DM).

### Cell culture

3T3-L1 preadipocytes (BCRC Number: 60159) were purchased from the Bioresource Collection and Research Center (Hsinchu, Taiwan). Cells were cultured with high glucose Dulbecco’s modified eagle medium (Gibco, Dublin, Ireland; 12100046), 10% fetal bovine serum (NQBB International Biological Corporation, Stockport, UK) and 1% Penicillin-Streptomycin (Gibco, Ireland; 15140122) at 37°C in a 5% CO_2_ incubator. After the cells reached confluence, the medium was changed to an adipogenic differentiation cocktail medium containing 0.5 mM 3-isobutyl-1-methylxanthine (IBMX, Sigma, St. Louis, MO, USA; I7018), 1 μM dexamethasone (Sigma, USA; D4902), and 10 μg/mL insulin (Sigma, USA; I1882) for 96 h. The medium was then changed to one containing 10 μg/mL insulin for a further 96 h. The medium was replaced every 48 h until the cells were fully differentiated on day 8.

### Cell viability assay

3T3-L1 preadipocytes were seeded in a 96-well plate (5×10^3^ cells/well) and incubated at 37°C and 5% CO_2_ for 24 h. After 24 h, the medium was changed to an adipogenic differentiation cocktail medium supplemented with JBP at various concentrations for 96 h. The medium was then changed to one containing 10 μg/mL insulin supplemented with JBP at various concentrations for a further 96 h. Once the medium was removed, PrestoBlue cell viability reagent (Invitrogen, Waltham, MA, USA; A13262) was added and the cells incubated for 1.5 h at 37°C. Absorbance was measured at 570 nm and 600 nm (the reference wavelength for normalization) with a microplate reader (Sunrise, Tecan, Switzerland), and the relative lipid content calculated as per the following formula:

$$\text{Cell viability }(\%)=({\text{A}}_{0}/{\text{A}}_{\text{1}})\times 100$$

where A_0_ is the absorbance of the sample and A_1_ is the absorbance of the blank.

### Oil red O staining and lipid content

To investigate the effects of JBP on lipid accumulation in 3T3-L1 preadipocytes, the cells were cultured for 8 days after the initiation of differentiation with various concentrations of JBP. After removing the medium, the cells were washed 2 times with phosphate-buffered saline (PBS) then fixed with 10% formalin for 30 min. The formalin was removed and cells were washed 2 times with PBS, before being stained with filtered oil red O working solution for 30 min. The oil red O working solution was then removed and cells were washed 4 times with distilled water. The stained lipid droplets were photographed under microscope. The oil red O stains on the cells were dissolved in isopropanol, and absorbance was measured at 540 nm with a microplate reader (Sunrise, Tecan, Switzerland). Relative lipid content was calculated as per the following formula:

$$\text{Relative lipid content }(\%)=({\text{A}}_{0}/{\text{A}}_{1})\times 100$$

where A_0_ is the absorbance of the sample and A_1_ is the absorbance of the blank.

### Triglyceride content

The intracellular triglyceride content was measured on day 8 after differentiation, as per the methods of Chen et al [13]. In brief, using a commercial triglyceride assay kit as per the manufacturer’s protocol (Sigma, USA; MAK040), absorbance was measured at 570 nm (reference wavelength for normalization) with a microplate reader (Sunrise, Tecan, Switzerland). Triglyceride (TG) content was calculated as per the following formula:

$$\text{TG content }(\text{mM})=({\text{S}}_{\text{a}}/{\text{S}}_{\text{v}})$$

where S_a_ is the amount of triglycerides in the unknown sample (nmoles) from the standard curve, and S_v_ is the sample volume (μL) added to the reaction well.

### Animal experiment design

A total of 320 one-day-old female red feather native chickens were evenly divided by weight, and then randomly assigned to one of 4 treatment groups for 12 weeks. All chickens were raised in a pen (2×4 square meters) with rice bran litter in a temperature-controlled house. There were 20 chickens per pen and 4 replicates per treatment for a total of 80 chickens per treatment group. The initial average body weight was 35±0.5 g (similar between all pens). The RT was set at 33°C ±1°C until the chickens were 7 days old, then decreased to 26°C±1°C day by day until the chickens were 21 days old. Thereafter, the RT was maintained at 26°C±1°C until the end of the experiment. The experiment was conducted at National Chung Hsing University in Taiwan and followed the protocols of the Animal Care and Use Committee (IACUC: 108-055).

### Feeding schedule and diet composition

The experimental period was divided into 3 phases: starter (days 1 to 28), grower (days 29 to 56), and finisher (days 57 to 84). Chickens were divided into 4 treatment groups: control, basal diet supplemented with 0.5% JBP (0.5% JBP), basal diet supplemented with 1% JBP (1% JBP), and basal diet supplemented with 5×10^6^ CFU/kg BA+5×10^6^ CFU/kg SC (BA+SC). Feed and water were provided *ad libitum*, and the diets were formulated to meet or exceed the nutritional requirements suggested by NRC (1994). The compositions of the starter, grower, and finisher basal diets are listed in Table 1.

### Sample collection and carcass characteristics

During the experiment, body weight and feed consumption were recorded on days 70 and 84, to determine body weight gain and feed conversion ratio (FCR). Blood samples were collected from brachial veins then stored at 4°C until further analysis. Six chickens of similarly average weight were selected from each treatment group and euthanized by electric shock on day 84. The liver, abdominal fat, and spleen were collected and stored in RNAzol reagent at −20°C for later analysis. From each treatment group, 5 chickens of similarly average weight fasted for 24 h, then were euthanized by exsanguination. The carcass and abdominal fat were weighed after depilation and the viscera were removed. Carcass percentage, abdominal fat percentage, and subcutaneous fat thickness were calculated, according to the methods of Zhang et al [14].

### Serum characteristics

Sixteen 84-day-old chickens (1 bird per pen, 4 birds per treatment) were randomly selected for blood sample collection. The blood samples were held at 4°C for 4 h, then centrifuged at 3,000 rpm for 15 min at 4°C. Serum biochemical parameters were analyzed with an automatic biochemical analyzer (Hitachi, 7150 auto-analyzer, Tokyo, Japan).

### Quantitative polymerase chain reaction analysis

The total mRNA of the chicken liver, abdominal fat, and spleen were isolated using RNAzol reagent (Molecular Research Centre, Inc., Cincinnati, OH, USA), according to the manufacturer’s protocols. The method of determination of total RNA concentration and purity, cDNA synthesis, and quantitative polymerase chain reaction (qPCR) analysis were modified from the method [15]. Normalization of gene expression data by internal control (β-actin) used the 2^−ΔΔCt^ method to calculate the relative mRNA expression level. The mean and standard deviation were calculated from sample data for the same treatment group. Gene-specific primers were designed based on the genes of Gallus gallus (chickens); these are listed in Table 2.

### Statistical analysis

Collected data were analyzed for significance by analysis of variance using the general linear model procedure in SAS software (SAS 9.4, 2018, SAS Institute Inc., Cary, NC, USA). Differences between treatment means were analyzed using Duncan’s multiple range test, and p<0.05 was considered significantly different.

## RESULTS

### Catechin composition and total phenol and flavonoid content

The bioactive compounds of different tea cultivar byproducts are shown in Table 3. This study analyzed and compared the catechin composition in the byproducts of oolong, Yingxiang, Jinxuan, Longjing, Biluochun, and black tea. All tea byproducts contained high levels of catechin except black tea, which exhibited the lowest amount of EGCG. Conversely, JBP had the highest amount of EGCG, while the main alkaloid was caffeine. As a result, JBP was chosen for further analysis; total phenol and flavonoid content were determined as 107.4 mg of GAE/g DM and 5.5 mg of QE/g DM, respectively (Table 4).

### Effect of Jinxuan byproduct on 3T3-L1 cell viability

**Figure 1:**
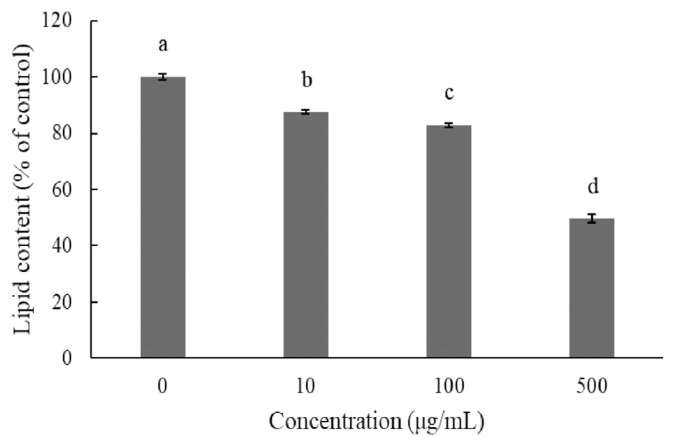
Effect of Jinxuan byproduct on 3T3-L1 cell viability during differentiation induction. ^a–d^ Means with different letters are significantly different (p<0.05).

The cytotoxic effects of JBP on cell viability are shown in Figure 1. The results show that there was a cytotoxic effect at high doses, and supplementation with JBP for 8 d reduced cell proliferation and viability. Cell viability significantly decreased with 500 μg/mL and 1,000 μg/mL.

### Effect of Jinxuan byproduct on adipogenic differentiation

**Figure 2:**
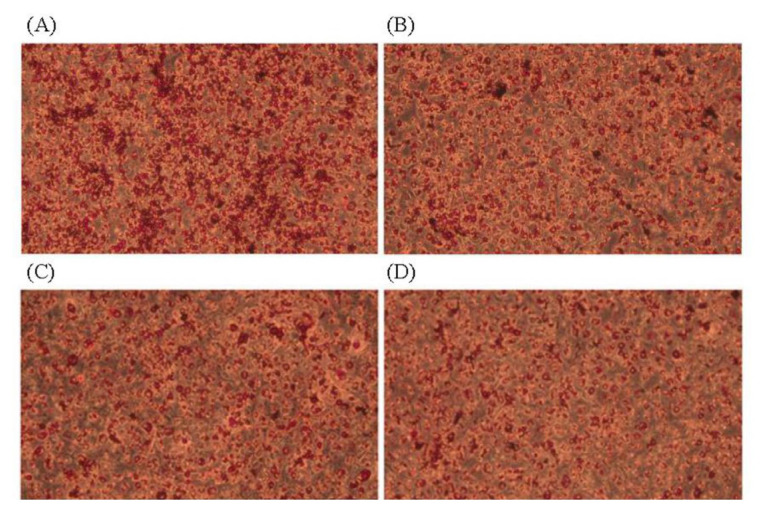
The 3T3-L1 adipocytes were stained with oil red O and the picture was taken under 20× lens magnification. (A) Control, (B) 10 μg/mL, (C) 100 μg/mL, (D) 500 μg/mL.

**Figure 3:**
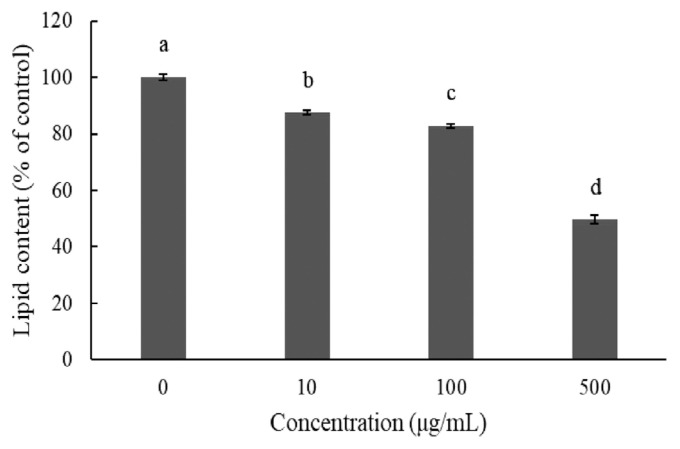
Effect of Jinxuan byproduct on lipids accumulation during 3T3-L1 pre-adipocytes differentiation. Post-differentiation, the lipid droplets were stained with oil red O (Magnification: 100×). (A) Control, (B) 10 μg/mL, (C) 100 μg/mL, (D) 500 μg/mL. ^a–d^ Means with different letters are significantly different (p<0.05).

Adipogenic differentiation of 3T3-L1 preadipocytes was investigated via oil red O staining in order to evaluate the anti-adipogenic effect of JBP (Figure 2). As oil red O only stains mature adipocytes, Figure 2 shows how stained oil droplets were reduced with JBP. Furthermore, the amount of intracellular lipid was significantly decreased with 10 μg/mL, 100 μg/mL, and 500 μg/mL JBP (Figure 3).

**Figure 4:**
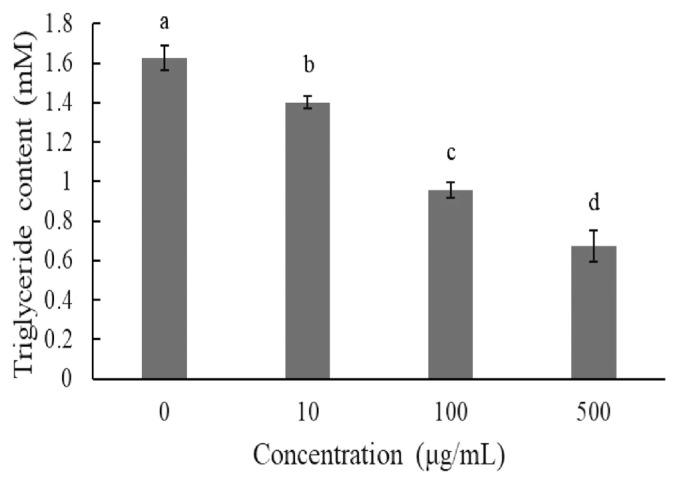
Effect of Jinxuan byproduct on lipids accumulation during 3T3-L1 pre-adipocytes differentiation. ^a–d^ Means with different letters are significantly different (p<0.05).

The TG content was also investigated on day 8 after induction. Supplementation with 10 μg/mL, 100 μg/mL, and 500 μg/mL of JBP significantly reduced TG content (Figure 4).

### Growth performance

The effects of dietary supplementation with JBP or BA+SC on the growth performance of red feather native chickens are shown in Table 5. FCR significantly improved in the 0.5% JBP and 1% JBP groups during days 1 to 56. Weight gain significantly increased in the 0.5% JBP and BA+SC groups during days 56 to 70. There were no significant differences among the groups during days 70 to 84 and 1 to 84.

### Serum biochemical parameters

Table 6 shows the effects of dietary supplementation with JBP or BA+SC on serum biochemical parameters in 84- day-old red feather native chickens. These include glucose, creatinine, uric acid, serum glutamic-oxaloacetic transaminase (SGOT), serum glutamic-pyruvic transaminase (SGPT), total protein, albumin, globulin, alkaline phosphatase, cholesterol (CHOL), TG, high-density lipoprotein cholesterol (HDL-C), and low-density lipoprotein cholesterol (LDL-C). None of the parameters were significantly different among the treatment groups.

### Carcass characteristics

The effects of dietary supplementation with JBP or BA+SC on the growth performance of red feather native chickens are shown in Table 7. Live weight, carcass weight, carcass percentage, abdominal fat percentage, and subcutaneous fat were not significantly different among the treatment groups.

### Selected gene expression in red feather native chickens

**Figure 5:**
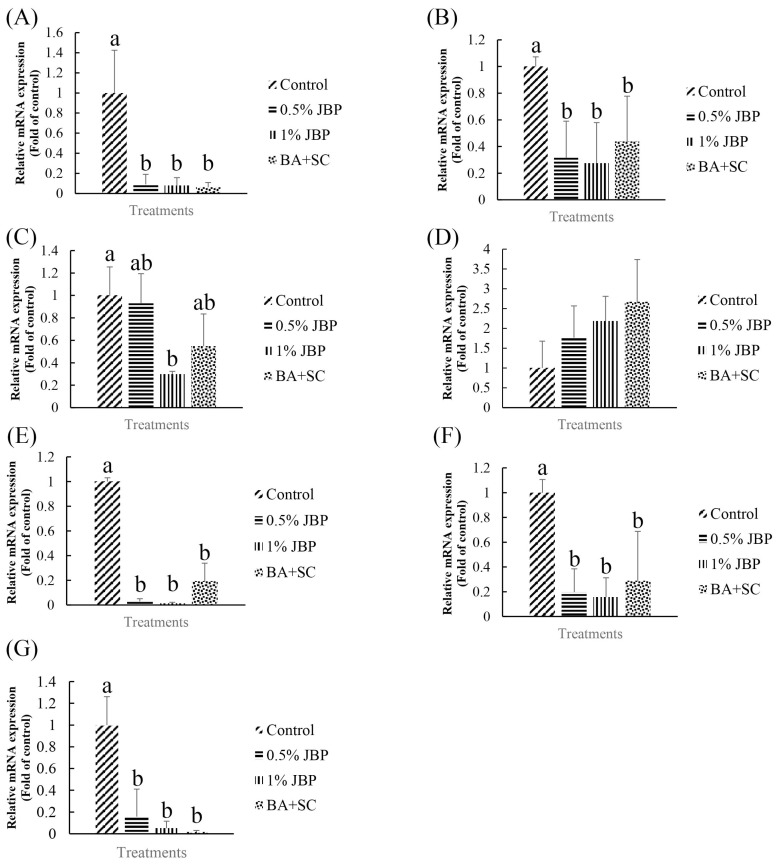
The mRNA expression level of (A) FAS, (B) ACC (by liver), (C) PPARγ, (D) LPL (by abdominal fat), and (E) NF-κB, (F) IL-1β, (G) TNF α (by spleen) in the red feather native chicken at 84 days. FAS, fatty acid synthase; ACC, acetyl-CoA carboxylase; PPARγ, peroxisome proliferator-activated receptor γ; LPL, lipoprotein lipase; NF-κB, nuclear factor kappa-light-chain-enhancer of activated B cells; IL-1β, interleukin 1 beta; TNF α, tumor necrosis factor alpha. Results are mean±standard deviation, and 6 birds per group. ^a,b^ Means with different letters are significantly different (p<0.05).

The effects of dietary supplementation with JBP or BA+SC on lipid metabolism and inflammation-related mRNA expression in 84-day-old red feather native chickens are shown in Figure 5. mRNA expression of fatty acid synthase (FAS) and acetyl-CoA carboxylase (ACC) in liver were significantly reduced in the 0.5% JBP, 1% JBP, and BA+SC groups compared to the control (p<0.05). In addition, the 1% JBP group had significantly decreased mRNA expression of peroxisome proliferator-activated receptor γ (PPAR γ) in abdominal fat compared to the control (p<0.05). Furthermore, nuclear factor kappa-light-chain-enhancer of activated B cells (NF-κB), interleukin 1 beta (IL-1β), and tumor necrosis factor alpha (TNF-α) in spleen were significantly decreased in the 0.5% JBP, 1% JBP, and BA+SC groups (p<0.05).

## DISCUSSION

Tea is one of the most consumed beverages in the world. It contains a large amount of phenolic compounds and catechins, especially green tea which has the highest amount of catechins. As such, green tea is considered to have many benefits, including anti-inflammation and anti-obesity effects in animals [2]. It has been reported that the quantity of catechins depends on the cultivar and its fermentation process [16]. In our study, we compared the byproducts of several tea cultivars (Table 3). JBP exhibited the highest amounts of catechins, especially the anti-obesity compound EGCG. Black tea possessed the lowest amount of catechins due to its manufacturing process. During fermentation, catechins in black tea are converted to theaflavins and thearubigins, decreasing the overall catechin content [17]. JBP also exhibited high amounts of phenols and flavonoids (Table 4). Based on these results, JBP has an abundance of bioactive compounds related to biological functionality, which have anti-obesity, anti-inflammatory, antibacterial, and antioxidative effects.

When excess energy is ingested, the excess energy is mainly stored as TG. Increases in adipose tissue mass lead to increased adipocyte size (hypertrophy) and number (hyperplasia). Both these and the inflammatory response are characteristic of obesity, which depends on the differentiation of preadipocytes [8]. In *in vitro* experiments, we determined that JBP supplementation has a dose effect on lipid content in 3T3-L1 cells. Higher concentrations of JBP reduce lipid content, however the highest concentration tolerated is 100 μg/mL. Similar results are shown in Figures 3 and 4, JBP supplementation can decrease 3T3-L1 cell differentiation thereby decreasing adipose accumulation. Previous studies have reported on the concentration-dependent inhibitory effects of EGCG on the differentiation of 3T3-L1 preadipocytes into mature adipocytes [8]. TG is one of the main substrates in lipid accumulation and JBP decreases TG accumulation in 3T3-L1 cells. Previous studies demonstrated that catechins could suppress the differentiation process by inhibiting the expression of PPAR γ and C/EBPα. Therefore, it appears that JBP could effectively decrease adipose accumulation and differentiation [18].

FCR was higher in the JBP-supplemented groups during days 1 to 56. This may be due to the slightly lower body weights and higher feed intake. The addition of JBP at early stages may increase energy consumption and fat metabolism, since the catechins and caffeine could have a negative effect on FCR [19]. In addition, green tea contains tannin, which could inhibit protease activity and may reduce protein digestibility, affecting the conversion of food to tissue [20]. The addition of JBP or probiotics did not seem to affect growth performance in red feather native chickens, which is similar to previous studies that showed that dietary supplementation with 0.5% to 1.5% green tea powder or 0.5% to 2% green tea byproduct had no significant effect on broilers [21,22].

The JBP and probiotics used were non-hepatotoxic, as shown in Table 6; no differences were observed in the serum levels of SGOT and SGPT. There were no differences among the groups in other serum parameters, including CHOL, TG, HDL-C, and LDL-C. These results were similar with Yang et al [22], which showed that dietary supplementation with green tea byproducts did not affect serum lipids in broiler chickens. Dietary supplementation with green tea powder had no significant impact on serum parameters [23], however Afsharmanesh and Sadaghi [9] demonstrated that green tea decreased serum lipids (total cholesterol and TG) and significantly increased HDL-C. In our study, there were no significant differences in carcass characteristics. Previous studies indicated that dietary supplementation with 1% green tea powder lowered the percentage of abdominal weight, but not enough to be significantly different from the control [24]. No differences were observed in carcass percentage or abdominal fat percentage [9]. However, in the study [21], dietary supplementation with 0.5% to 1.5% Japanese green tea powder significantly decreased abdominal fat percentage without having a significant impact on carcass weight or percentage. In addition, oral administration of 40 mg/kg or 80 mg/kg body weight EGCG per day significantly decreased abdominal fat percentage in broilers [25]. Green tea powder and EGCG have different impacts on the percentage of abdominal fat, however the previously mentioned study that directly administered green tea powder did not determine catechin composition, or the level of EGCG in particular. Therefore, it is difficult to directly compare the effects of green tea powder supplementation in broilers. Conversely, this study compared catechin composition and used JBP in its animal experiments, which contains the highest amount of EGCG among the tested tea byproducts. While 1% JBP slightly decreased abdominal fat percentage, it was not significantly different from the control. Differences in environment, diet, cultivar, and tea processing may explain these discrepancies, especially the latter two as they may strongly affect EGCG content [23].

Liver and adipose tissue are the two main sites of *de novo* fatty acid synthesis in higher vertebrates [8]. However, Leveille et al [26] suggested that chicken adipose tissue was of minor importance compared to the liver, accounting for less than 30% of total fatty acid synthesis. As such, in contrast to other mammals, the major site of fatty acid synthesis in chickens is the liver, rather than adipose tissue. PPAR γ, FAS, and ACC are all lipogenesis-related mRNA that induce fat accumulation. Of those, FAS and ACC are the key enzymes in *de novo* fatty acid synthesis [27]. PPAR γ participates in the early stages of adipocyte differentiation, while FAS is induced during late stages [28]. PPAR γ is a major adipogenic transcription factor, mainly expressed in adipose tissue, and plays a key role in adipocyte differentiation. It is highly expressed in the adipose tissue of fatty chickens, which is the major site of lipogenesis in avian species, and induces the expression of adipocyte-related proteins like FAS and ACC [29]. Plasma lipoproteins are hydrolyzed by lipoprotein lipase (LPL); low expression levels or the absence of LPL leads to marked lipemia and triglyceridemia. Obesity is accompanied by chronic inflammation due to an increased production of pro-inflammatory cytokines like TNF-α and IL-1β, which are activated by NF-κB [30]. In this study, lipogenesis-related mRNA levels in 84-day-old chickens were significantly decreased in both the JBP and BA+SC supplemented groups. The 1% JBP group in particular showed a significant decrease in the expression levels of PPAR γ. There was no significant difference in LPL among the groups. Aside from its effect on adipose metabolism-related mRNA expression, it is well known that inflammation is a double-edged sword in animals. While the inflammatory response can help in the defense against pathogens, it can also cause cell damage if there’s an overreaction. Furthermore, as adipocytes secrete low amounts of cytokines, excess adipocytes in animals will cause low grade inflammation. The current study shows that JBP could suppress adipocyte differentiation and reduce adipocyte numbers if EGCG levels are high, which could in turn decrease the inflammatory response. Moreover, it seems that both JBP and BA+SC supplementation could decrease NF-κB, IL-1β, and TNF-α mRNA expression, which are all inflammation-related upstream mRNA and cause the further secretion of cytokines. Decreasing adipose accumulation with JBP and BA+SC could suppress both lipogenesis- and inflammation-related mRNA expression. There appeared to be no significant differences in carcass characteristics between the groups, possibly because the amount of JBP supplementation was not high enough or because the changes in fat metabolism were not abdominal or subcutaneous. Further research is needed to clarify this interaction.

## CONCLUSION

In the current study, the catechin compositions of different tea cultivar byproducts were compared, and their anti-obesity ability analyzed by *in vitro* 3T3-L1 preadipocyte tests. Dietary supplementation with JBP or BA+SC resulted in no significant reduction in abdominal fat in chickens, but JBP or BA+SC could modulate lipid metabolism and the inflammatory response. Moreover, a practical use for the massive amount of byproduct created by the tea industry is an added benefit.

## Figures and Tables

**Table 1:** Ingredients and chemical composition of the experimental diets for red feather native chickens

Items	g/kg		

	Starter diet (1 to 28 days)	Grower diet (29 to 56 days)	Finisher diet (57 to 84 days)
Ingredients			
Corn, yellow	554.0	595.0	688.0
Soybean meal (CP 44%)	232.8	227.9	163.3
Full fat soybean meal	100.0	100.0	71.4
Soybean oil	35.3	16.2	15.3
Monocalcium phosphate	11.2	9.8	10.0
Calcium carbonate	10.0	19.0	19.4
DL-methionine	1.4	0.8	0.7
NaCl	2.0	3.0	3.1
Choline-Cl	0.8	0.8	0.8
Vitamin premix1)	1.0	1.0	1.0
Mineral premix2)	1.0	1.0	1.0
Total	1,000	1,000	1,000
Calculated nutrient value			
ME (kcal/kg)	3,200	3,000	3,142
Crude protein (%)	23	20	18.10
Calcium (%)	0.87	1.08	1.08
Total phosphorus (%)	0.72	0.62	0.61
Available phosphorus (%)	0.48	0.38	0.38
Lysine (%)	1.36	1.14	1.00
Methionine (%)	0.55	0.42	0.39
Methionine+cystein (%)	0.90	0.74	0.69
Threonine (%)	0.74	0.68	0.61
Cl (%)	0.07	0.05	0.05
Na (%)	0.05	0.04	0.04
K (%)	0.83	0.76	0.67

ME, metabolizable energy.

1) Supplied per kg of diet: Vit A 15,000 IU; Vit D_3_ 3,000 IU; Vit E 30 mg; Vit K_3_ 4 mg; riboflavin 8 mg; pyridoxine 5 mg; Vit B_12_ 25 μg; Ca-pantothenate 19 mg; niacin 50 mg; folic acid 1.5 mg; biotin 60 μg.

2) Supplied per kg of diet: Co (CoCO_3_) 0.255 mg; Cu (CuSO_4_·5H_2_O) 10.8 mg; Fe (FeSO_4_·H_2_O) 90 mg; Zn (ZnO) 68.4 mg; Mn (MnSO_4_·H_2_O) 90 mg; Se (Na_2_SeO_3_) 0.18 mg.

**Table 2:** Characteristics and performance data of the primers used for quantitative polymerase chain reaction analysis

Genes	Primers	Oligonucleotide sequence (5′-3′)	Accession in GenBank
β-actin	F	5′-CTGGCACCTAGCACAATGAA-3′	X00182.1
	R	5′-ACATCTGCTGGAAGGTGGAC-3′	
PPARγ	F	5′-GATCGCCCAGGTTTGTTAAA-3′	NM_001001460
	R	5′-TGCACGTGTTCCGTTACAAT-3′	
FAS	F	5′-GCTGAGAGCTCCCTAGCAGA-3′	NM_205155
	R	5′-TCCTCTGCTGTCCCAGTCTT-3′	
ACC	F	5′-TGTGGCTGATGTGAGCTTTC-3′	NM_205505
	R	5′-ACTGTCGGGTCACCTTCAAC-3′	
LPL	F	5′-TGAGGGAATCGAGAGCAAGT-3′	NM_205282
	R	5′-TACATTCCTGTCACCGTCCA-3′	
NF-κB	F	5′-GAAGGAATCGTACCGGGAACA-3′	NM_205134
	R	5′-CTCAGAGGGCCTTGTGACAGTAA-3′	
IL-1β	F	5′-GCTCTACATGTCGTGTGTGATGAG-3′	NM_204524
	R	5′-TGTCGATGTCCCGCATGA-3′	
TNF α	F	5′-CCGAGGAGTACCCCAAAGAC-3′	MF000729.1
	R	5′-TGATACAGCGACTCGAACCA-3′	

PPARγ, peroxisome proliferator activated receptor gamma; FAS, fatty acid synthase; ACC, acetyl-CoA carboxylase; LPL, lipoprotein lipase; NF-κB, nuclear factor of kappa light polypeptide gene enhancer in B-cells p65; IL-1β, interleukin 1-beta; TNF α, tumor necrosis factor alpha.

**Table 3:** The content of bioactive compounds in different tea by-product

Items (mg/g DM)	Oolong	Yingxiang	Jinxuan	Longjing	Biluochun	Black tea
Gallic acid	0.63±0.01	0.28±0.02	0.91±0.01	0.16±0.03	0.17±0.03	0.23±0.01
Gallocatechin	1.85±0.02	1.67±0.02	1.61±0.01	1.47±0.04	1.19±0.02	0.14±0.01
Epigallocatechin	31.4±0.61	22.9±1.09	25.7±0.31	10.7±0.33	9.78±1.47	1.02±0.07
Catechin	1.49±0.04	1.17±0.03	2.04±0.03	0.56±0.13	0.69±0.08	0.08±0.00
Epicatechin	4.75±0.13	3.63±0.20	3.39±0.03	2.16±0.14	1.49±0.20	0.11±0.04
Epigallocatechin gallate	45.4±0.70	33.5±1.78	52.5±0.60	18.6±0.95	18.1±2.17	0.26±0.03
Gallocatechin gallate	0.66±0.01	0.38±0.03	0.64±0.01	0.27±0.16	0.18±0.01	0.30±0.07
Epicatechin gallate	3.70±0.14	2.71±0.18	3.72±0.04	1.63±0.14	1.22±0.24	0.18±0.00
Caffeine	16.2±0.10	17.0±0.91	23.9±0.24	10.0±0.40	7.73±1.01	0.06±0.00

All data are expressed as means±standard deviation from three replicates (n = 3).

DM, dry matter.

**Table 4:** The content of total phenolic and flavonoid in Jinxuan byproduct

Items	Jinxuan byproduct
Total phenolic (mg of GAE/g DM)	107.4±1.9
Total flavonoid (mg of QE/g DM)	5.5±0.1

All data are expressed as means±standard deviation from three replicates (n = 3).

GAE, gallic acid equivalent; DM, dry matter; QE, quercetin equivalent.

**Table 5:** The effect of Jinxuan byproduct supplemented in diets on growth performance of 1 to 84 d-old red feather native chickens

Items	Experimental diets1)				SEM	p-value

	Control	0.5% JBP	1% JBP	BA+SC		
1–56 d						
Body weight (g)2)	1,912	1,735	1,755	1,837	20.9	0.057
Feed consumption (g)2)	4,439	4,629	4,634	4,362	58.8	0.318
Weight gain (g)2)	1,877	1,700	1,720	1,802	20.9	0.056
FCR2	2.37^b^	2.72^a^	2.69^a^	2.42^b^	0.03	0.007
57–70 d						
Body weight (g)	2,387	2,457	2,343	2,470	16.0	0.054
Feed consumption (g)	1,505	1,631	1,494	1,718	57.4	0.471
Weight gain (g)	475^b^	722^a^	588^ab^	633^a^	20.9	0.019
FCR	3.17	2.26	2.54	2.71	0.09	0.235
71–84 d						
Body weight (g)	2,870	2,893	2,860	3,033	43.9	0.517
Feed consumption (g)	2,020	1,733	1,651	1,976	123.8	0.702
Weight gain (g)	483	436	517	563	44.1	0.771
FCR	4.18	3.97	3.19	3.51	0.13	0.150
1–84 d						
Feed consumption (g)	7,964	7,993	7,779	8,056	93.2	0.671
Weight gain (g)	2,835	2,858	2,825	2,998	43.9	0.517
FCR	2.81	2.80	2.75	2.69	0.04	0.669

JBP, Jinxuan byproduct; SEM, standard error of the mean; FCR, feed conversion ratio; CFU, colony-forming unit.

1) Control, basal diet; 0.5% JBP, basal diet supplemented with 0.5% JBP; 1% JBP, basal diet supplemented with 1% JBP; BA+SC, basal diet supplemented with 5×10^6^ CFU/kg *Bacillus amyloliquefaciens*+5×10^6^ CFU/kg *Saccharomyces cerevisiae*.

2) Each value represents the mean of 4 replicates (20 birds in each replicate).

a,b Means within the same rows with different letters are significantly different (p<0.05).

**Table 6:** The effect of JBP supplemented in diets on serum characteristics of 84d-old red feather native chickens

Items	Experimental diets1)				SEM	p-value

	Control	0.5% JBP	1% JBP	BA+SC		
GLU (mg/dL)	210	221	237	247	5.37	0.137
CREA (mg/dL)	0.14	0.09	0.10	0.09	0.02	0.656
UA (mg/dL)	4.55	5.65	5.80	5.43	0.35	0.608
SGOT (U/L)	242	219	224	206	9.28	0.610
SGPT (U/L)	6.00	4.50	3.75	4.00	0.32	0.112
TP (g/dL)	4.73	4.58	4.33	4.13	0.16	0.577
ALB (g/dL)	2.00	1.98	1.98	1.75	0.07	0.536
GLO (g/dL)	2.73	2.60	2.35	2.38	0.10	0.518
Alk-P (IU/L)	906	1,028	752	1,002	84.29	0.657
CHOL (mg/dL)	148	156	158	143	4.05	0.525
TG (mg/dL)	92.8	124.5	112.0	140.0	13.97	0.682
HDL-C (mg/dL)	82.8	88.5	85.3	84.5	2.04	0.792
LDL-C (mg/dL)	62.5	66.3	69.0	56.8	2.61	0.414

Each value represents the mean of four replicates (n = 4).

JBP, Jinxuan byproduct; SEM, standard error of the mean; GLU, glucose; CREA, creatinine; UA, uric acid; SGOT, glutamic-oxalocetic transaminase; SGPT, serum glutamic-pyruvic transaminase; TP, total protein; ALB, albumin; GLO, globulin; ALK-P, alkaline phosphatase; CHOL, cholesterol; TG, triglycerides; HDL-C, cholesterol-high-density lipoprotein; LDL-C, cholesterol-low-density lipoprotein; CFU, colony-forming unit.

1) Control, basal diet; 0.5% JBP, basal diet supplemented with 0.5% JBP; 1% JBP, basal diet supplemented with 1% JBP; BA+SC, basal diet supplemented with 5×10^6^ CFU/kg *Bacillus amyloliquefaciens*+5×10^6^ CFU/kg *Saccharomyces cerevisiae*.

**Table 7:** The effect of Jinxuan byproduct supplemented in diets on carcass characteristics of 84 d-old red feather native chickens

Items	Experimental diets1)				SEM	p-value

	Control	0.5% JBP	1% JBP	BA+SC		
Live weight (g)	2,628	2,660	2,570	2,636	23.1	0.574
Carcass weight (g)	2,208	2,201	2,141	2,182	22.7	0.726
Carcass percentage (%)	84.0	82.8	83.3	82.8	0.27	0.340
Abdominal fat (%)	1.69	1.69	1.42	1.56	0.06	0.338
Subcutaneous fat (mm)	5.43	4.79	6.20	4.87	0.21	0.098

Each value represents the mean of four replicates (n = 4).

JBP, Jinxuan byproduct; SEM, standard error of the mean; CFU, colony-forming unit.

1) Control, basal diet; 0.5% JBP, basal diet supplemented with 0.5% JBP; 1% JBP, basal diet supplemented with 1% JBP; BA+SC, basal diet supplemented with 5×10^6^ CFU/kg *Bacillus amyloliquefaciens*+5×10^6^ CFU/kg *Saccharomyces cerevisiae*.
